# Structured and prompt treatment of early arthritis in clinical practice leverages window of opportunity and leads to excellent clinical outcomes: an innovative retrospective cohort study

**DOI:** 10.1007/s10067-024-07192-z

**Published:** 2024-10-29

**Authors:** R. L. Teixeira, R. da Silva Vieira, M. J. Saavedra, J. Polido-Pereira, R. A. Moura, I. Alcobia, J. E. Fonseca, V. C. Romão

**Affiliations:** 1 Rheumatology Department, Unidade Local de Saúde de Santa Maria, Centro Académico de Medicina de Lisboa, Av. Prof. Egas Moniz MB, 1649-028 Lisbon, Portugal; 2 Faculdade de Medicina, Universidade de Lisboa, Centro Académico de Medicina de Lisboa, Av. Prof. Egas Moniz MB, 1649-028 Lisbon, Portugal

**Keywords:** Arthritis, Quality of life, Referral and consultation, Rheumatic diseases

## Abstract

**Objectives:**

With this work, we evaluated the impact of the Lisbon Early ARthritis cliNic (LEARN) on untreated inflammatory arthritis clinical and patient-reported outcomes.

**Methods:**

A retrospective cohort study enrolled patients in LEARN since its inception. Patients were followed for 12 months and treated to achieve disease remission. Clinical, structural, and quality of life outcomes were assessed. The early arthritis module of the Portuguese Rheumatic Diseases Registry (Reuma.pt) is described.

**Results:**

We assessed 292 patients between 2015 and 2022. Mean symptom duration and DAS-28-4 V-ESR at baseline were 6.2 ± 3.5 months and 5.6 ± 1.3, respectively. Rheumatoid arthritis (56.4%; 40.1% seropositive) and psoriatic arthritis (12.4%) were the most common diagnoses. Most patients were treated with methotrexate (75.3%) combined with low-dose oral prednisolone (88.1%). At 12 months, a mean ΔDAS28-4 V-ESR improvement of 2.3 ± 0.4 was registered, with 29.5% and 48.9% of patients achieving remission (DAS28-4 V-ESR < 2.6) or low disease activity (DAS28-4 V-ESR < 3.2), respectively. Among RA patients only, these figures were 20.6% and 46.6%, respectively. A clinically meaningful functional improvement was observed in 72.1% of the patients. Structural progression was limited, affecting only 16.1% of the patients. Fatigue, anxiety, depression, and quality of life also improved substantially, translated by improvements in FACIT, HADS, EQ5D, and SF-36 scores.

**Conclusions:**

A structured, dedicated approach to patients with early arthritis resulted in good clinical, structural, and functional outcomes. Furthermore, our findings suggest the window of opportunity for early intervention may have implications for mental health and global well-being.

**Table:** 

**Key Points** • *Patient assessment is facilitated by reliable electronic clinical records, such as the early arthritis module of the Rheumatic Diseases Portuguese Register (Reuma.pt) which we describe here for the first time.* • *Inflammatory arthritis was confirmed in the majority of patients observed, but the time to first appointment was above the recommended.* • *Prompt start of conventional therapy allowed significant disease activity improvement and remission to be achieved in about one-third of the patients.* •* Key patient-reported outcomes elucidate disease impact and confirm the benefit of early treatment initiation, suggesting a window of opportunity also for mental health and global well-being. *

**Supplementary Information:**

The online version contains supplementary material available at 10.1007/s10067-024-07192-z.

## Introduction

Clinically arthritis is the most common feature of the early phase of inflammatory rheumatic diseases. During the first months, many patients show diverse articular involvement, with morning stiffness, joint swelling, pain, and significant functional impairment [1]. Rheumatoid arthritis (RA) is the most common diagnosis in patients presenting with chronic persistent symmetrical polyarthritis. Symptom duration has been identified as a main prognostic factor in achieving clinical remission, thus defining a ‘window of opportunity’ to treat patients effectively [2]. Early arthritis is also a common feature in spondylarthritis (SpA), which can affect peripheral joints, usually in a different pattern than RA, in addition to the axial skeleton and entheses. In psoriatic arthritis (PsA), the effectiveness of a treat-to-target approach with tight clinical control has been demonstrated in high-quality randomised controlled trials [3]. Furthermore, inflammatory arthralgia and RA-like polyarthritis are among the most common early manifestations of several connective tissue diseases (CTD), which often remain undiagnosed for long periods of time [4].

This manuscript aims to highlight the importance and added value of well-structured management of patients presenting with early arthritis. We describe our early arthritis outpatient clinic and emphasise the use and singularities of the early arthritis module within the Rheumatic Diseases Portuguese Register (Reuma.pt) [5]. A complete characterisation of the early arthritis cohort is made, detailing demographic data, clinical presentation, serologic profile, diagnosis, treatment modality, and outcome.

## Patients and methods

### Lisbon Early ARthritis cliNic (LEARN)

Santa Maria Hospital, within the Lisbon Academic Medical Center (CAML), is a tertiary university hospital and research campus that receives patients from all over Portugal, mainly the southern region. The Rheumatology Department provides more than 30,000 appointments and 20,000 procedures per year. From 2015 onwards, we have established a dedicated outpatient clinic aimed at the care of patients presenting with peripheral arthritis for less than 12 months. It receives patients referred from the general practitioner (GP), different departments within the hospital, including the emergency department (ED), and other hospitals. The dedicated team is made up of two rheumatologists, who do both the initial and subsequent assessments during the first 12 months of follow-up. When a patient is referred, he/she is booked for the early arthritis clinic team within a maximum of 7 days. Every week, 3 new patients are observed, plus up to 5 patients under follow-up. If patients are referred with urgent criteria, additional appointments are arranged.

### Patient evaluation and registry

**Fig. 1:**
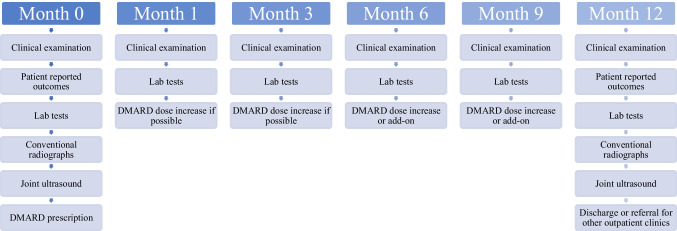
Patient follow-up flowchart and clinical procedures

Shortly after the referral, patients are evaluated by a trained rheumatologist and included in the cohort if inflammatory arthralgia and joint tumefaction are confirmed. Patients with suspected crystal deposition or septic arthritis are excluded. No classification criteria were used in this real-world approach. Early arthritis patients have an appointment scheduled as described in Fig. 1—baseline evaluation before disease-modifying anti-rheumatic drug (DMARD) start and follow-up at 1, 3, 6, 9, and 12 months (Fig. 1a). After that period, patients are directed to the general rheumatology clinic or other specific clinics (e.g., RA, PsA, SpA, systemic lupus erythematosus (SLE), Sjögren’s disease (SjD), among others) depending on the clinical diagnosis established. This allows for a high patient turnover while maintaining easy access to new referrals.

The Reuma.pt early arthritis dedicated module is the basis for the electronic clinical record used in this clinic. The Portuguese Society of Rheumatology created Reuma.pt in June 2008. It works as an electronic medical record and is available across all Portuguese rheumatology centres. It allows the prospective collection of clinical and laboratory data for patient follow-up and systematic verification of classification criteria [5].

A lefthand side menu can be used to collect general and disease-specific items (supplementary Fig. 1). It includes identification, clinical synopsis, occupational context and literary habilitations, date of symptom onset and diagnosis, as well as tobacco and alcohol consumption habits. Arthritis-related variables such as arthralgia, its rhythm and topography, morning stiffness, and duration are all collected. Extra-articular manifestations are also registered, including fever, oral ulcers, Raynaud’s phenomenon, sicca symptoms, photosensitivity, diarrhoea, psoriatic lesions, and urethral or vaginal exudate, among others. Current and previous pharmacological and non-pharmacological treatments, procedures, and surgeries are registered. A gynecologic and obstetric history can be obtained and reported in female patients. A specific early arthritis panel of key laboratory results is available, including erythrocyte sedimentation rate (ESR), C-reactive protein (CRP), antinuclear antibodies (ANA), rheumatoid factor (RF), anti-cyclic citrullinated peptides (anti-CCP), and uricemia. Complement and specific autoantibodies can also be registered.

For each appointment, different indexes are offered to capture disease activity and impact. A homunculus (supplementary Fig. 2) is used to record tender and swollen joints. Along with these, patient (PtGA) and physician (PhGA) global assessment visual analogue scales (VAS; 0–100), ESR, and CRP values are registered. Disease activity indexes are automatically calculated, providing immediate disease activity score-28 joints (DAS-28), clinical disease activity index (CDAI), and simple disease activity index (SDAI), allowing an easy graphic longitudinal view of the data [6].

If patients present enthesitis, a homunculus with 16 enthesitis points (supplementary Fig. 2b) is available, immediately providing an enthesitis score, such as the Maastricht Ankylosing Spondylitis Enthesitis score (MASES), Leads Enthesitis Index (LEI), and Spondyloarthritis Research Consortium of Canada (SPARCC) [7–9]. Patients with axial involvement can directly fill in patient-reported outcomes (PROs) such as the Bath Ankylosing Spondylitis Disease Activity Index (BASDAI) or the Ankylosing Spondylitis Disease Activity Score (ASDAS, with current CRP) [10, 11]. Of note, both indexes are interlinked, connecting the overlapping responses filled in.

Some patients may evolve into different connective tissue diseases such as SLE or SjD. Key clinical tools such as the SLE Disease Activity Index 2000 (SLEDAI-2 K) [12], EULAR Sjögren’s Systemic Disease Activity Index (ESSDAI) [13], and EULAR Sjögren’s Patient-Reported Index (ESSPRI) [14] are available to assess disease activity and impact when applicable.

A group of patient-reported outcomes (PROs) are accessible. The Health Assessment Questionnaire–Disability Index (HAQ-DI) assesses physical disability and the daily impact of arthritis and disease damage [15]. The 5-dimensional EQ-5D-5L, developed by the EuroQol Group, is used to measure health-related quality of life [16]. A validated version of short form-36 (SF-36) is also used [17, 18]. Mental health status can be characterised using the Hospital Anxiety and Depression Scale (HADS) [19]. Finally, chronic fatigue is assessed by the Functional Assessment of Chronic Illness Therapy (FACIT) Fatigue Scale, version 4 [20]. All questionnaires are applied in validated Portuguese versions.

### Imaging

Radiographs of the hands, feet, and other involved joints are obtained at baseline. Joint erosions are actively sought and recorded. The same set of radiographs is repeated at 12 months or earlier if clinically indicated and evaluated by a trained rheumatologist. A baseline chest radiographic evaluation is obtained in all patients. Patients undergo articular ultrasound assessment of disease activity, which may assist in differential diagnosis when clinically needed. Hands and wrists are systemically screened for joint effusion, bone erosions, and tenosynovitis, as well as any structural findings, as defined by EULAR-OMERACT [21–24]. When indicated, an ultrasound of other affected joints is performed. A Canon Aplio® i800 and GE Logiq® S7 ultrasound machines are used routinely and handled by five trained ultrasonographers.

### Laboratory evaluation and early arthritis biobanking

Peripheral blood is collected to complement clinical observation, as described above. Systematic evaluation includes complete blood count, ESR, CRP, serum creatinine, aspartate/alanine aminotransferases, urinalysis, ANA, RF, anti-CCP, as well as hepatitis B, hepatitis C, and HIV1/2 antibodies. Further specific tests are requested to aid in the differential diagnosis, namely extractable nuclear antigen tests when a CTD is suspected. Moreover, upon specific informed consent, additional blood samples are collected to obtain DNA, serum, and peripheral blood mononuclear cells. All samples are kept frozen at Biobanco-IMM, CAML, for subsequent research projects [25]. A specific early arthritis biobank collection was created to allow for longitudinal sample collection.

### Governance and ethics

The patient authorises all data collection through informed consent, as approved by the CAML Ethics Committee with the approval number being 138/19. Reuma.pt is approved by the Portuguese National Commission for Data Protection and by the local ethics committees. All identifiable data is encrypted and only accessible through an individual password. Each clinician can only visualise and edit data registered in his/her own centre. Biobanco-IMM, CAML is approved by the National Commission for Personal Data Protection [25]. Patient management is conducted according to the World Medical Association (WMA) Declaration of Helsinki, as amended in Fortaleza in 2013 [26].

### Statistical analysis

Demographic and clinical characteristics are presented as frequency and mean (*x̄*) ± standard deviation (SD). Comparison of continuous variables is performed using student *t*-test (for normal distributed values, two groups), ANOVA (for normal distributed values, three or more groups), Mann–Whitney *U*-test (for non-normal distributed values, two groups) or Kruskal–Wallis (for non-normal distributed values, three or more groups). Categorical variables were compared using Chi-square or Fisher’s exact test. Statistical analysis was conducted using Office Excel 2016 for Windows (Microsoft, USA) and GraphPad-Prism-8.0.2 for Windows (GraphPad Software, USA). Statistical significance was defined as *p* < 0.05.

## Results

### Patient characteristics

Between 2015 and 2022, a total of 292 patients were evaluated. Most patients were referred by the general practitioner (*n* = 176, 60.3%); 60 (20.5%) originated from the emergency department, 38 (13.0%) from different medical or surgical specialties within our centre, and 18 (6.2%) from other external outpatient clinics. Out of 292 patients, 202 (69.2%) were confirmed to have inflammatory arthritis with less than 12 months of symptomatic duration (Fig. 1b). Almost one-third of patients (*n* = 90, 30.8%) were discharged after the first evaluation due to the absence of immune-mediated (*n* = 58) or early (i.e., ≥ 12 months of symptoms; *n* = 18) arthritis. Demographic and presenting clinical features of the 202 included patients are presented in Table 1.

**Table 1:** Early inflammatory arthritis cohort demographic and baseline clinical features

	*n* = 202
Age (years), mean ± SD	57.8 ± 16.0
Female, *n* (%)	142 (70.3)
Caucasian, *n* (%)	160 (79.2)
Smoking history, *n* (%)	
Active	38 (20.7)
Previous	26 (14.1)
Never	120 (65.2)
Obesity [BMI > 30], *n* (%)	34 (23.0)
Arterial hypertension	90 (44.6)
Diabetes mellitus (type II)	39 (19.3)
Dyslipidaemia	65 (32.2)
Chronic kidney disease	3 (1.5)
Ischemic heart disease	8 (4.0)
Cerebrovascular disease	10 (5.0)
Interstitial lung disease	3 (1.5)
Obstructive lung disease	16 (8.0)
Cancer (except non-melanoma skin cancer)	23 (11.4)
Symptom duration (months), mean ± SD^1^	6.2 ± 3.5
Rheumatoid factor positive, *n* (%)	83 (41.1)
Anti-cyclic citrullinated peptide, *n* (%)	80 (39.9)
Antinuclear antibody positive, *n* (%)	35 (21.5)
Radiographic erosions, *n* (%)	
Hand	23 (11.4)
Feet	11 (5.4)
Presentation pattern,* n* (%)	
Polyarticular	106 (52.5)
Oligoarticular	82 (40.6)
Monoarticular	14 (7.0)
Tender joint count (TJC), mean ± SD	8.3 ± 6.3
Swollen joint count (SJC), mean ± SD	6.2 ± 4.8
Erythrocyte sedimentation rate (ESR) (mm/1st h), mean ± SD	50.5 ± 32.2
C-reactive protein (CRP) (mg/dL), mean ± SD	2.1 ± 2.5
Patient Global Assessment (PtGA) (VAS, 0–100)	65.1 ± 20.0
DAS28-4 V-ESR, mean ± SD	5.6 ± 1.3

^1^Range, 2 weeks–12 months

*BMI* body mass index

### Patient diagnosis

During the 12-month follow-up time, most patients (198, 98.0%) met clinical and serological features of specific rheumatic diseases (Table 2). The majority of the patients had a diagnosis of RA (56.4%), 68% of whom fulfilled the ACR/EULAR 2010 classification criteria. The second most common diagnosis was PsA, with or without enthesitis. All polymyalgia rheumatica (PMR) patients presented with clinically detectable arthritis, hence the referral to this clinic. Although microcrystalline etiologies were to be excluded at baseline, some patients had an initial suspicion of immune-mediated early arthritis, and the diagnosis of calcium pyrophosphate crystal deposition (CPPD) disease was established later on.

**Table 2:** Distribution of patients according to final diagnosis

Diagnosis	Patients, *n* (%)
Rheumatoid arthritis	
RF/anti-CCP positive	81 (40.1)
RF/anti-CCP negative	33 (16.3)
Psoriatic arthritis	25 (12.4)
Polymyalgia rheumatica	9 (4.5)
Systemic lupus erythematosus	6 (3.0)
Sjögren’s disease	3 (1.5)
Systemic sclerosis	1 (0.5)
Anti-synthetase syndrome	1 (0.5)
Mixed connective tissue disease	5 (2.5)
Viral arthritis	6 (3.0)
Reactive arthritis	1 (0.5)
Calcium pyrophosphate dihydrate crystal deposition	7 (3.5)
Osteoarthritis	12 (6.0)
Undifferentiated arthritis	10 (5.0)
RS3PE	1 (0.5)
Hemochromatosis	1 (0.5)

### Patient follow-up and therapy

Patients were started on conventional synthetic DMARD (csDMARD) therapy as soon as they were received at the early arthritis outpatient clinic, usually starting with methotrexate (*n* = 152, 75.3%; Table 3), with dose escalation up to the maximum tolerated. Steroid prescription followed the “lowest dose for shortest amount of time” rule.

**Table 3:** Disease activity class according to DAS28-4 V-ESR at baseline and follow-up

Patients, *n* (%)			
Disease activity class	Baseline (*n* = 201)	Month 6 (*n* = 190)	Month 12 (*n* = 139)
HDA	124 (61.7)	0 (0)	6 (4.3)
MDA	71 (35.3)	112 (70.9)	65 (46.8)
LDA	3 (1.5)	18 (11.4)	27 (19.4)
Remission	3 (1.5)	28 (17.2)	41 (29.5)

HDA, high disease activity, defined as DAS28 > 5.1; MDA, moderate activity, defined as DAS28 between 3.2 and 5.1; LDA, low activity, defined as DAS28 between 2.6 and 3.2; remission as defined by DAS28 < 2.6

A total of 48 patients were lost to follow-up, nine of whom in the first 3 months (Fig. 1b). Causes of death were myocardial infarction (an 88-year-old male with seronegative RA, under methotrexate (15 mg/week) and prednisolone (7.5 mg/day)); and SARS-CoV-2 pneumonia in the early pandemic period (a 90-year-old obese and unvaccinated female with psoriatic arthritis treated with methotrexate (20 mg/week) and sulfasalazine (2 g/day). In nine cases, there was methotrexate intolerance, which was switched to leflunomide. Hydroxychloroquine and/or sulfasalazine were added throughout the follow-up period for disease control in 26 (12.9%) and 19 (9.4%) patients, respectively. Most patients (88%) were concomitantly treated with low-dose oral prednisolone. Drug treatment, namely the number of patients who received each drug, mean, and cumulative doses as well as time on each dose, is detailed in Supplementary Table 1.

No patient needed to start a biologic (bDMARD) or target synthetic DMARD (tsDMARD) during the first 12 months. However, five patients were proposed for b/tsDMARD therapy at the end of the follow-up.

### Clinical and imaging outcomes

**Fig. 2:**
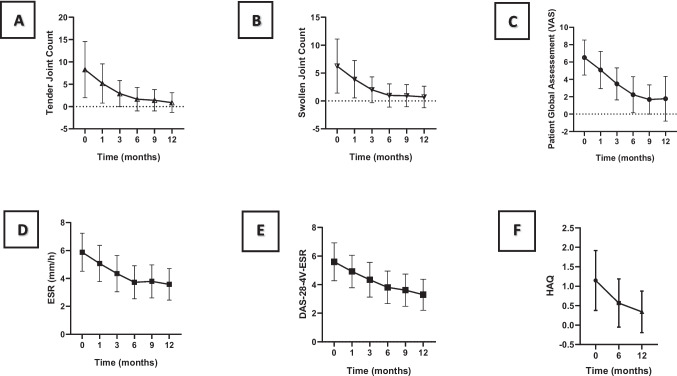
Disease activity and physical function during the 12-month follow-up. **A** Tender joint count, **B** swollen joint count, **C** patient global assessment, **D** erythrocyte sedimentation rate (ESR), **E** disease activity score 4-variables ESR, **F** health assessment questionnaire (HAQ)

Disease activity parameters improved significantly during the follow-up, as shown by the reduction of tender and swollen joint counts, inflammatory markers, and patient global assessment (Fig. 2).

The whole cohort had a significant decrease in DAS28-4 V-ESR over time, corresponding to a ΔDAS28-4 V-ESR of 2.3 ± 0.4. CDAI and SDAI also fell significantly (supplementary Fig. 3). Of note, 41 patients (29.5%) achieved remission (given by DAS28-4 V-ESR < 2.6), while a total of 68 patients (48.9%) attained a DAS28-4 V-ESR < 3.2 at 12 months (Table 3).

When considering only RA patients (*n* = 116), DAS28-4 V-ESR fell from 5.8 ± 1.2 to 3.4 ± 1.0. Also, a decrease of CDAI from 27.6 ± 12.6 to 5.2 ± 7.2 and SDAI from 29.6 ± 13.6 to 5.8 ± 7.6 was observed (supplementary Fig. 4A). At 12 months, a total of 20.6% of RA patients achieved remission, with 46.6% attaining a DAS28-4 V-ESR < 3.2 (supplementary Table 2A). Moreover, when considering only PsA patients (*n* = 25), DAS28 fell from 5.3 ± 1.4 to 3.1 ± 1.2. CDAI and SDAI also fell significantly, from 23.9 ± 9.5 to 4.2 ± 3.9 and 25.9 ± 10.6 to 4.7 ± 4.1, respectively (supplementary Fig. 4B). Regarding RA patients, a continuous disease activity reduction since the first month of DMARD therapy was noted, with a stabilisation between month 9 and 12, while PsA patients took a longer time to respond and then achieved lower disease activity indices (supplementary Fig. 4A + B). In fact, the proportion of PsA patients achieving remission at 12 months was 23.5%, with 52.9% attaining a DAS28-4 V-ESR < 3.2, higher percentages than within the RA sub-cohort (supplementary Table 2B).

Furthermore, the reduction in disease activity was paralleled by a significant functional improvement, with reductions in HAQ-DI from 1.15 ± 0.77 at baseline to 0.57 ± 0.62 and 0.34 ± 0.53 at months 6 and 12, respectively, with a ΔHAQ of 0.93 ± 0.20 (Fig. 2f). A clinically meaningful functional improvement (ΔHAQ-DI ≥ 0.22) was observed in 72.1% of the patients. Considering structural damage, 11.4% of the patients presented with radiographic hand erosions at baseline, which progressed to 15.3% after 12 months. Foot erosive disease was present in 5.4% at baseline and 7.4% at the end of the follow-up. As expected, patients with RA had a higher proportion of hand and foot erosions at baseline (*n* = 16, 14.8%) and after one year (*n* = 23, 16.1%).

### Mental health and quality of life outcomes

**Fig. 3:**
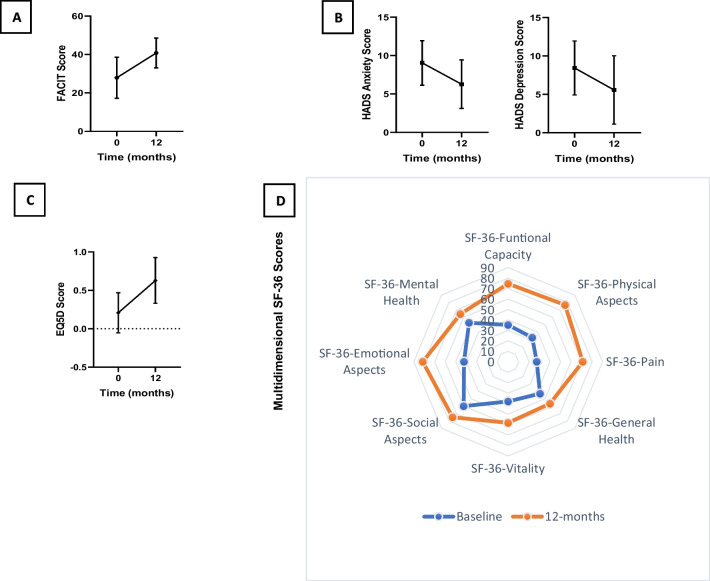
Fatigue, anxiety, depression, and quality of life, characterised by a set of patient-reported outcomes at the beginning and the end of the 12-month follow-up. **A** Functional assessment of chronic illness therapy—fatigue scale (FACIT); **B** hospital anxiety and depression scores (HADS); **C** EQ5D; **D** short form health survey-36 (SF-36)

A subgroup of patients (*n* = 80) had available data on fatigue, mental health, and quality of life. In this sample, fatigue significantly improved, translated by a rise in FACIT scores from 27.9 ± 10.7 at baseline to 40.8 ± 7.6 at 12 months (Fig. 3a). This aspect was most prominent among seropositive RA patients, which had a FACIT score of 22.4 ± 8.4 at baseline and an improvement to 42.5 ± 5.8 at 12 months. Anxiety and depression scores (HADS) improved significantly over time. While anxiety scores fell from 9.0 ± 2.9 to 6.3 ± 3.2, depression scores dropped from 8.4 ± 3.5 to 5.6 ± 4.6 (Fig. 3b). Patients with significantly high scores (≥ 11) [27] were mostly female (84.4%). Likewise, health-related quality of life improved substantially, with an increase in EQ-5D of 67% (Fig. 3c). The most significant improvements were found in physical and pain domains of SF-36, followed by physical function and mental health (Fig. 3d; supplementary Table 3).

## Discussion

Our manuscript highlights the importance of an early arthritis clinic to ensure the best comprehensive clinical and imaging outcomes. This cohort showed a mean symptom duration at enrolment of 6.2 ± 3.5 months, which is similar to previous studies. However, only a limited proportion of patients (16.3%) was observed within the recommended first 6 weeks of symptom onset [1]. A similar cohort recently described shorter times (< 4 months) between symptom onset and the first rheumatology appointment [28]. However, only 65.7% of the patients had a diagnosis of an immune-mediated disease, and among these, just 58.9% had less than 12 months of symptoms. There are discrepancies among patients in the time mediating general practitioners’ first observation and the referral. These observations reflect different thresholds for referral, which can delay the first rheumatology appointment. Also, many patients lack health literacy to understand which symptoms should prompt medical attention. These are the greatest factors explaining variation between 6 weeks and 12 months of symptom duration.

Barriers to this patient pathway have been previously identified [29]. We have implemented support for facilitating patient access [30], providing prompt help to GPs in the diagnosis and referral. Further collaborative efforts are nonetheless required to increment public and clinician awareness, improve peer-to-peer communication, and develop algorithms to support medical decisions.

In our cohort, more than half of the patients had a final diagnosis of RA (56.4%), followed by PsA (12.4%) and other connective tissue diseases (9.0%). These proportions are consistent with previous results from our group, in which, among very early arthritis patients, within the first 6 weeks of disease onset, 57% evolved into RA [31]. These data differ slightly from other existing cohorts, which usually have a higher proportion of RA patients (65–100%) and are focused on identifying individuals with a high probability of RA, excluding other rheumatic conditions [32–34].

Interestingly, patients in our clinic had higher baseline disease activity (mean DAS28 5.6 ± 1.3) than most other published cohorts (supplementary Table 3). Disease activity reduced significantly throughout follow-up, especially during the first months of treatment, a finding consistent with prior studies [35–37]. A ΔDAS28 of 2.3 ± 0.4 is an important indicator of disease amelioration, proving to be above values achieved in different cohorts reporting conventional practice, instead of a more intensive, DAS-driven approach in the context of treat-to-target trials. Achievement of remission is discrepant among the published cohorts, and comparisons can only be made between RA cohorts. Among our RA patients, 20.6% achieved remission at 12 months, which is comparable to that of a British observational study (21%) and to the TICORA trial (16%) in the conventional practice arm [38, 39]. Also, 46.6% of the RA patients attained a DAS28-4 V-ESR < 3.2, similar to other published cohorts, such as PEAC (40.2%) and ESPOIR (43.8%) [40, 41].

The observed prednisolone cumulative and mean doses are below the ones described in ESPOIR (cumulative dose of 3316 ± 1842 mg and mean 8.6 ± 8.5 mg) but are more than double compared to the PEAC study (cumulative dose of 1050 ± 819 mg and mean of 3.7 ± 2.7 mg). This reflects differences in geographical and local clinical practice. On the other hand, methotrexate use and doses were comparable to clinical-practice-based cohorts [40, 41]. The use of other drugs, such as hydroxychloroquine, sulfasalazine, and leflunomide, was similar to that described in the ESPOIR cohort, while increased use is described by the Leiden cohort and PEAC [40, 42].

Interestingly, only 11.4% of our patients had erosive disease at baseline, while among the RA patients, the proportion was raised to 14.8%. Both are below the 22% in ESPOIR and 23% in the Scottish cohort [33, 36]. This suggests discrepancies in referral and recruiting practices or may potentially reflect milder disease severity in a Southern European population.

Our cohort showed a significant decrease in the HAQ, with most (72.1%) patients achieving a minimal important clinical difference. Overall, this finding is consistent with previously published cohorts (supplementary Table 3) and underlines the notion of disability closely related to disease activity in early disease. [36, 43]

The disease's impact on mental health and quality of life is poorly studied in the available literature and has been seldom described. A recent study including 838 patients showed that RA patients had worse mental health outcomes than patients with pre-RA and undifferentiated arthritis [44]. We confirmed the findings on fatigue, with most of our patients having high FACIT scores at baseline, particularly RA patients. Also, it is known that anxiety has been reported more significantly than depression symptoms, which might be related to significant initial functional burden and disbelief about a possible recovery. As in other cohorts, female patients had worse baseline anxiety and depression scores, the reason for this not being entirely understood. [44, 45] Quality of life is significantly impaired from the beginning and throughout the disease course. In our cohort, both seropositive and seronegative RA showed worse quality of life than PsA, as assessed by SF-36. Of interest, a Swedish study reported significantly better quality of life as assessed by SF-36 in patients with early RA compared to patients with established disease, especially in women [45]. Taken together, these results suggest that early treatment intervention might improve mental health and global well-being outcomes.

This study has limitations, namely related to patient recruitment and data collection. While we continually try to optimise patients’ pathways in our department, some patients might have not been seen in the early arthritis clinic. They may have been treated directly, bypassing the early arthritis outpatient clinic, where data collection often competes with time limitations. Also, some missing data prevented further analysis, in terms of disease characterisation, activity, impact, and treatment. In addition, the missing data due to patients lost to follow-up and the reliance on observed data without accounting for potential confounders may affect the generalizability of the findings.

Finally, some difficulties persist when instituting a true treat-to-target approach due to safety concerns and failed adherence. We expect to improve these aspects soon by reinforcing the collaboration between dedicated rheumatologists, associated nurses, and informed patients.

Our study highlights the importance of a structured, dedicated approach to patients with early arthritis. It allows for early disease recognition and prompt treatment start, resulting in good clinical, structural, and functional outcomes. Furthermore, our findings suggest that the window of opportunity for early intervention also has positive implications for mental health and global well-being.

## Supplementary Information

Below is the link to the electronic supplementary material.

Supplementary file1 (PPTX 1696 KB)
