# Supplementary material for: Structured and prompt treatment of early arthritis in clinical practice leverages window of opportunity and leads to excellent clinical outcomes: an innovative retrospective cohort study
Source: Clin Rheumatol. 2024 Oct 29;43(12):3941–50. doi: 10.1007/s10067-024-07192-z (PMC11582134; doi:10.1007/s10067-024-07192-z)
Supplement: Supplementary file 1 — Supplementary file1 (PPTX 1696 KB) [file 10067_2024_7192_MOESM1_ESM.pptx]

## Slide 1
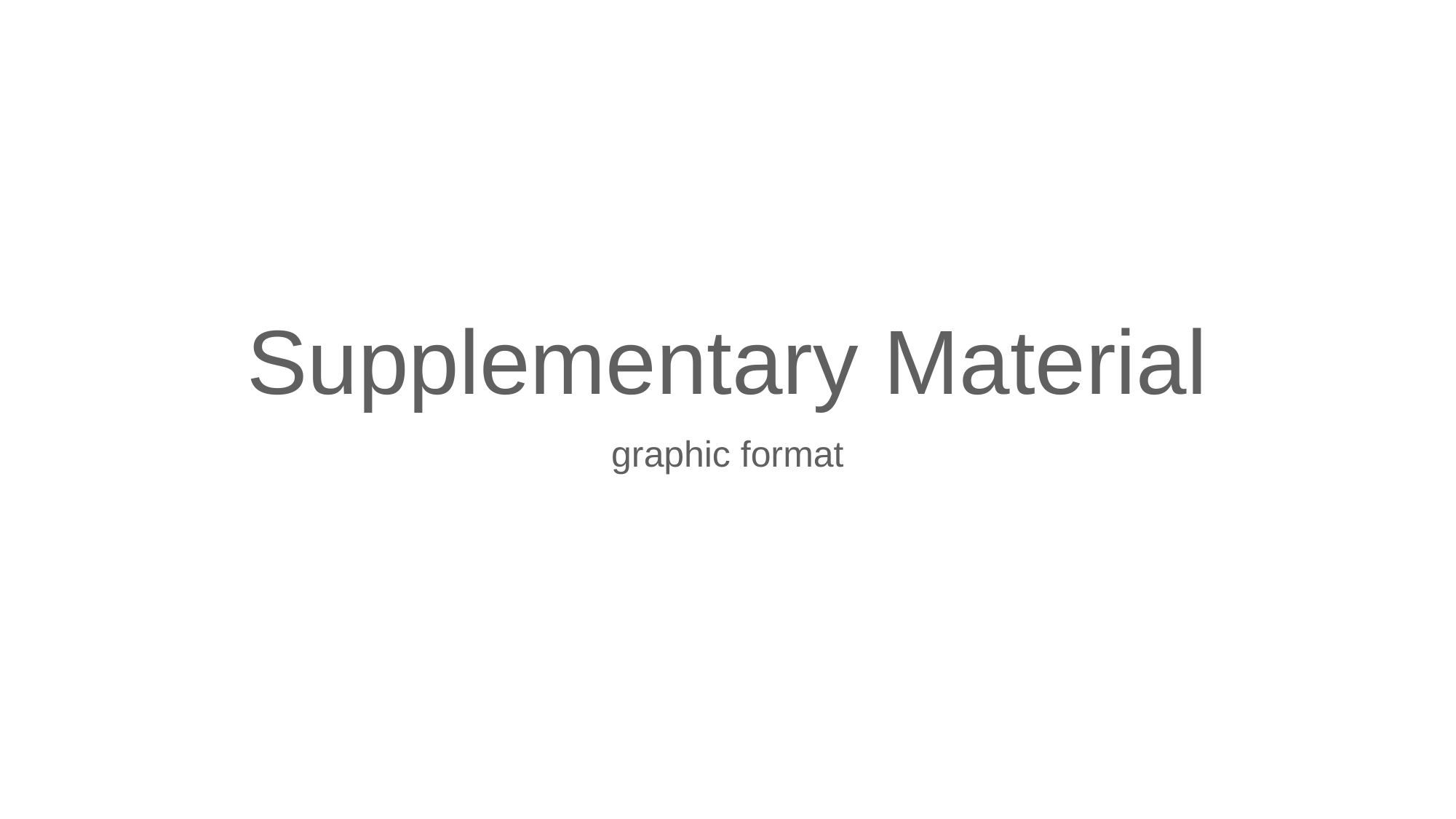

# Supplementary Material
graphic format

## Slide 2
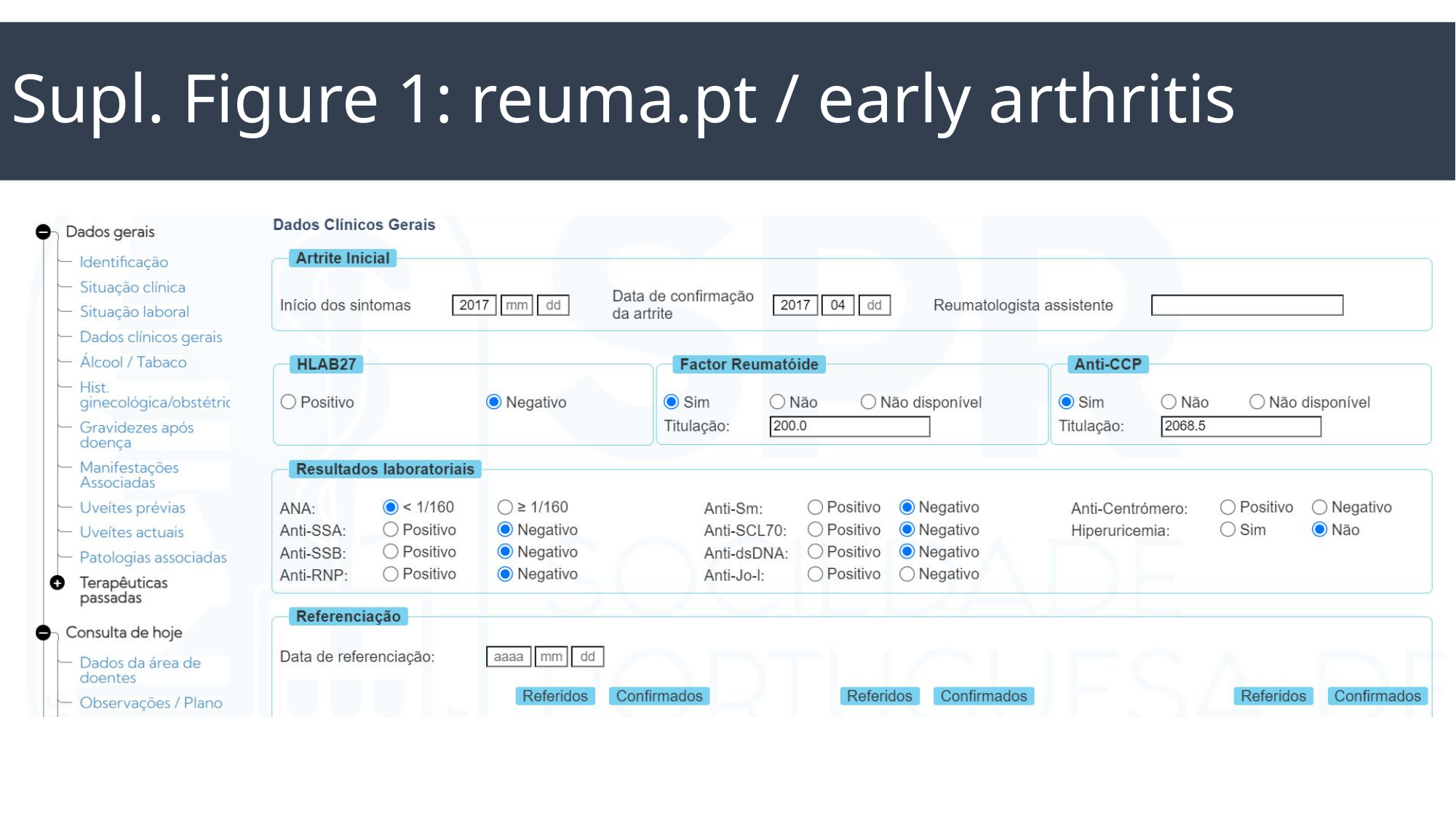

Supl. Figure 1: reuma.pt / early arthritis

## Slide 3
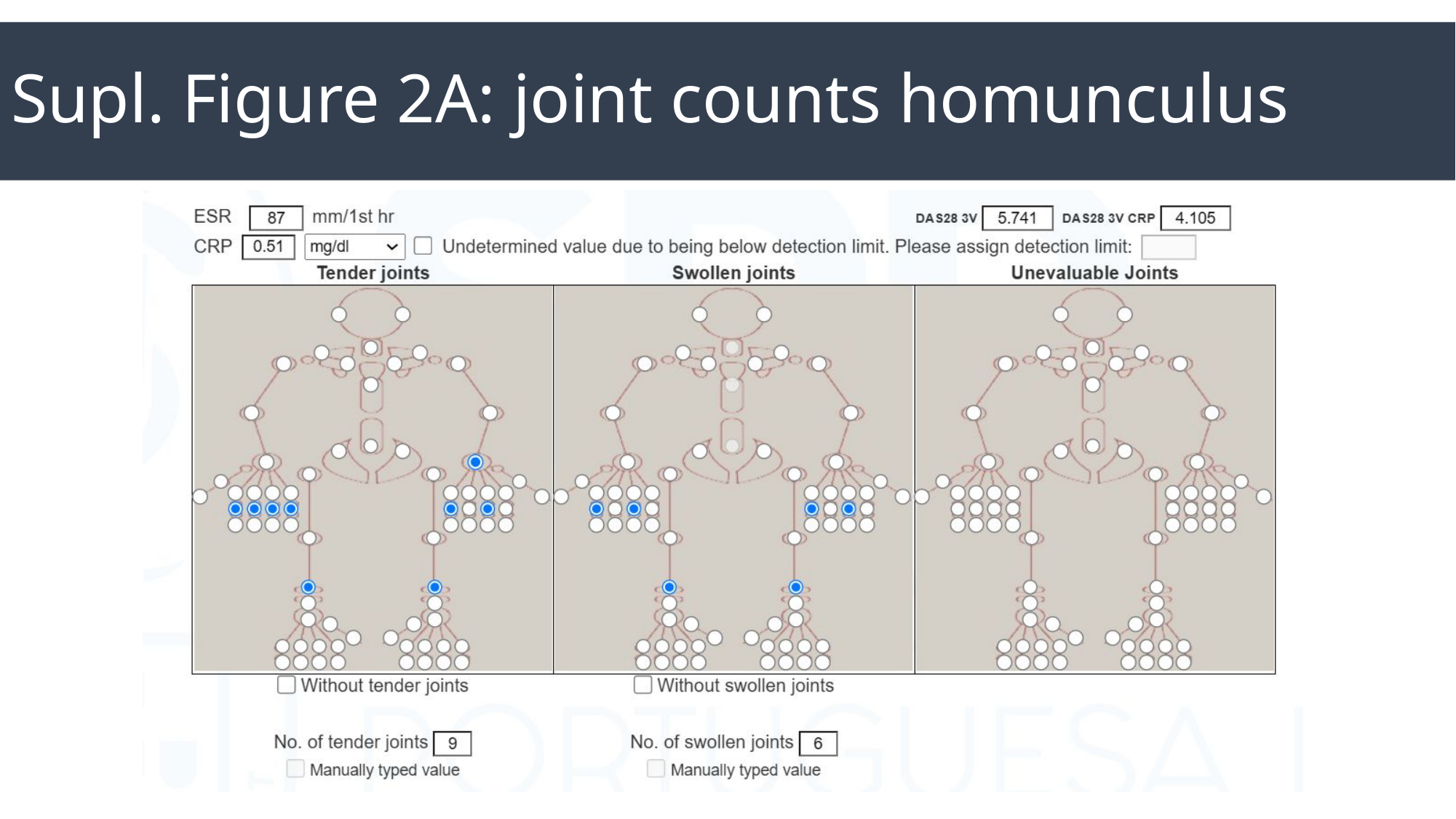

Supl. Figure 2A: joint counts homunculus

## Slide 4
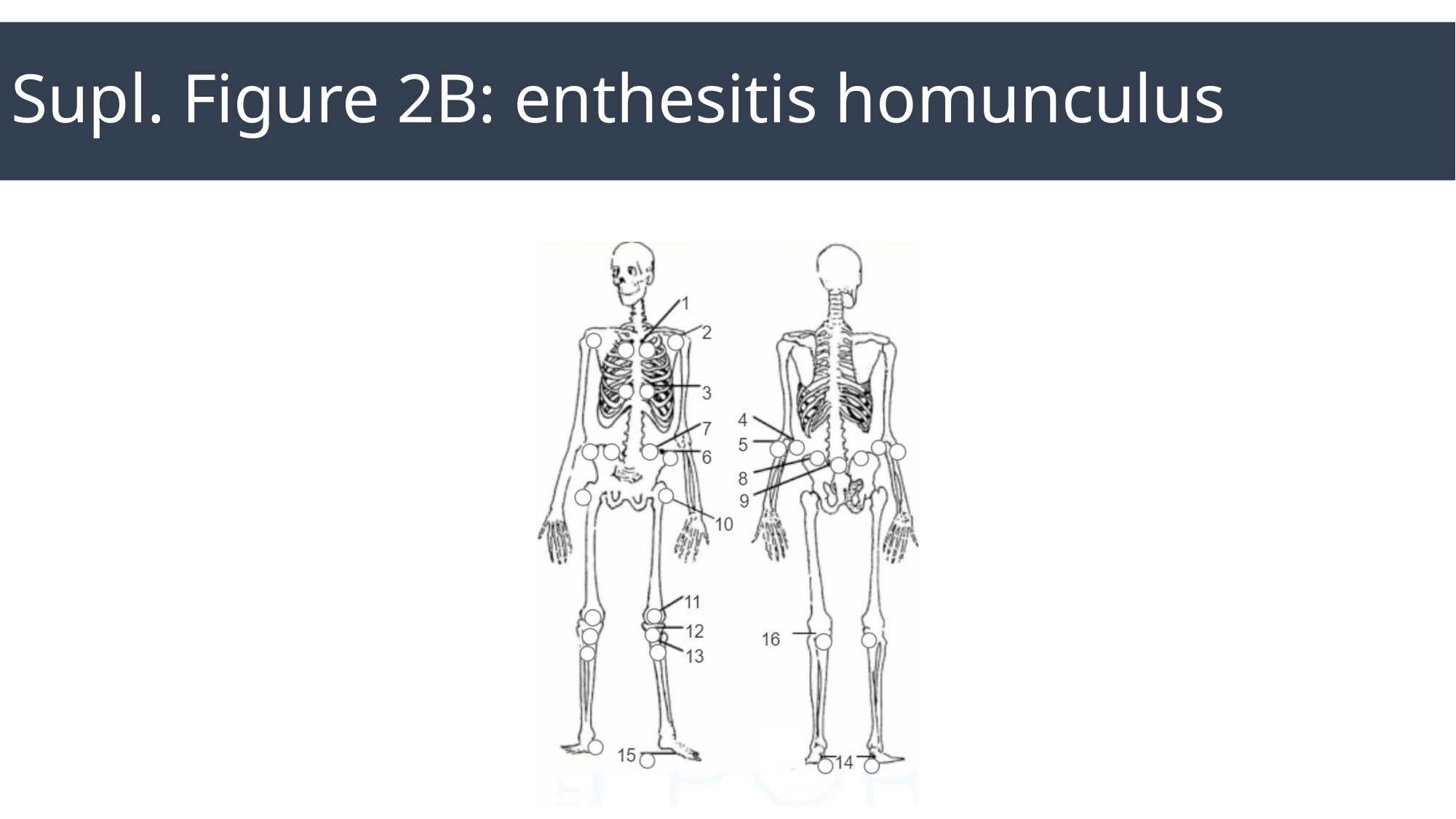

Supl. Figure 2B: enthesitis homunculus

## Slide 5
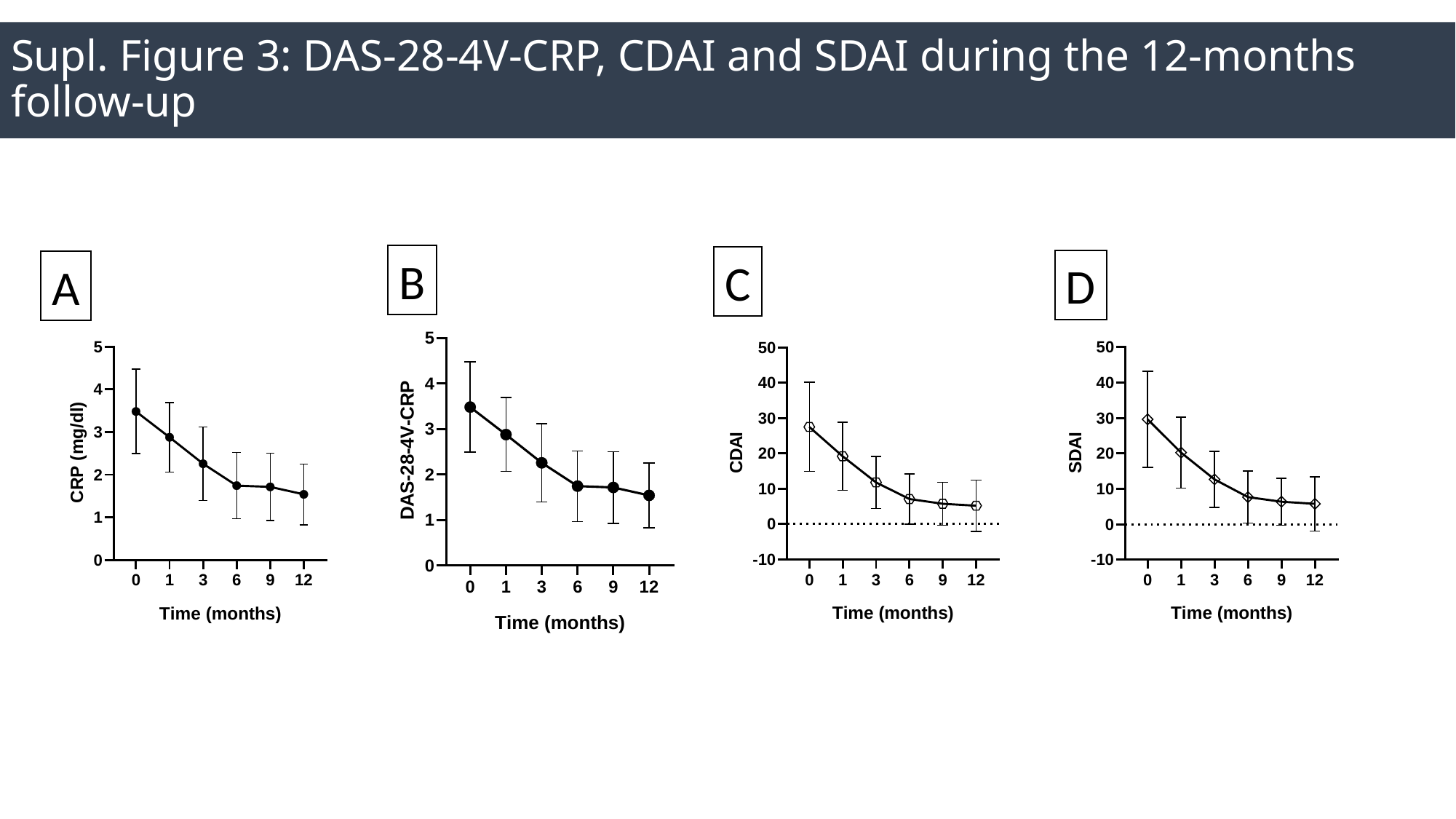

Supl. Figure 3: DAS-28-4V-CRP, CDAI and SDAI during the 12-months follow-up
B
C
D
A

## Slide 6
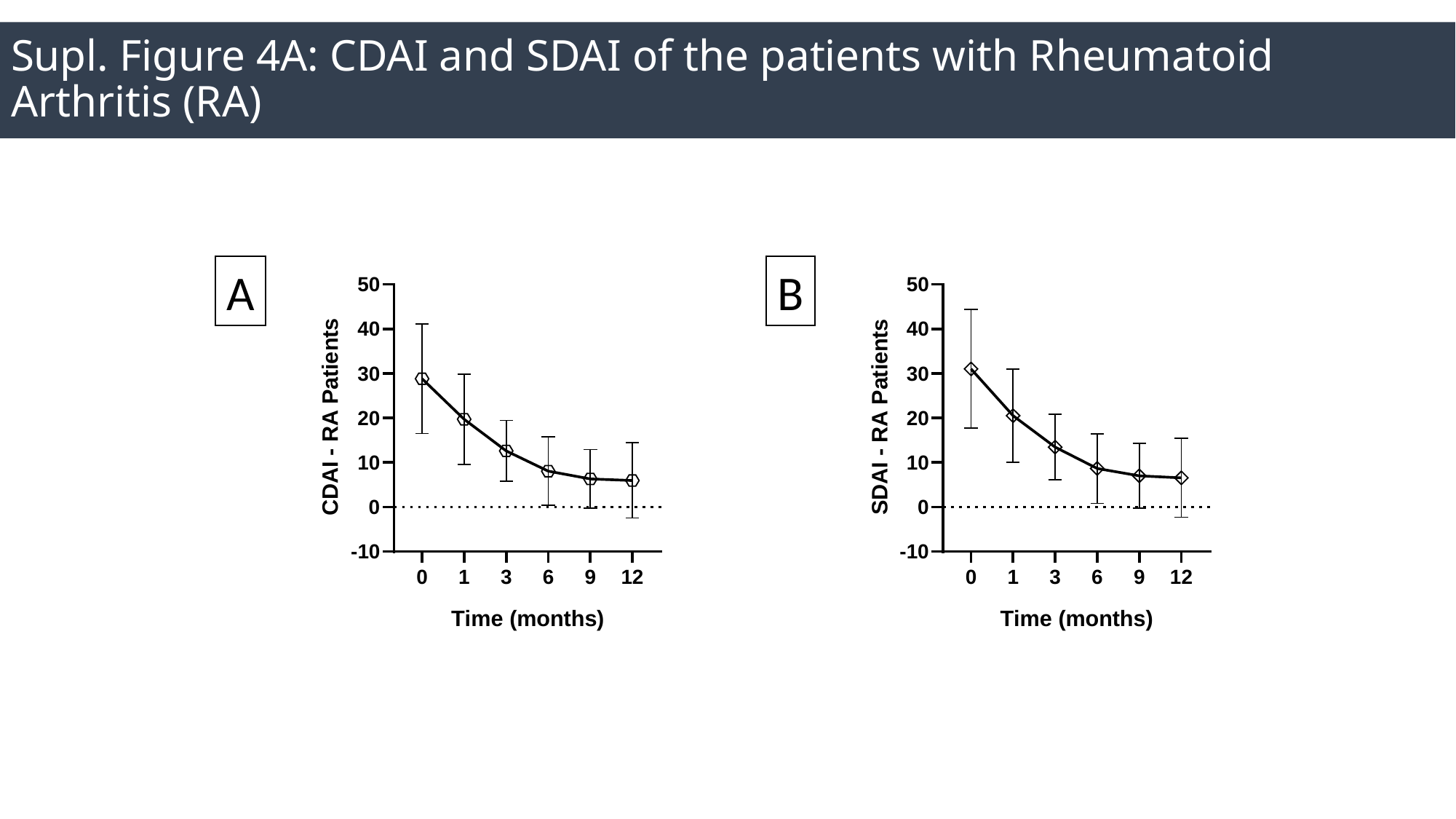

Supl. Figure 4A: CDAI and SDAI of the patients with Rheumatoid Arthritis (RA)
A
B

## Slide 7
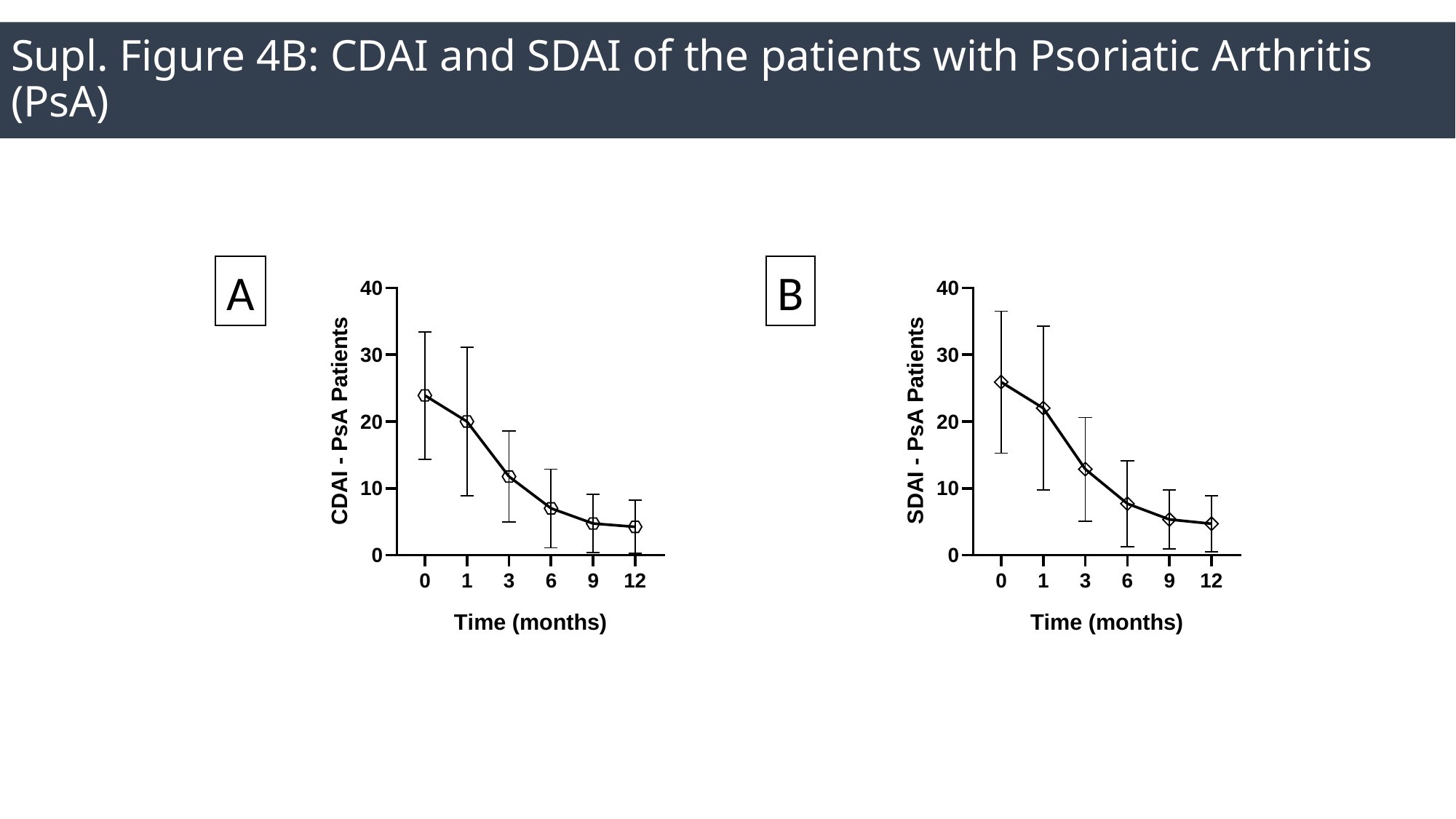

Supl. Figure 4B: CDAI and SDAI of the patients with Psoriatic Arthritis (PsA)
A
B

## Slide 8
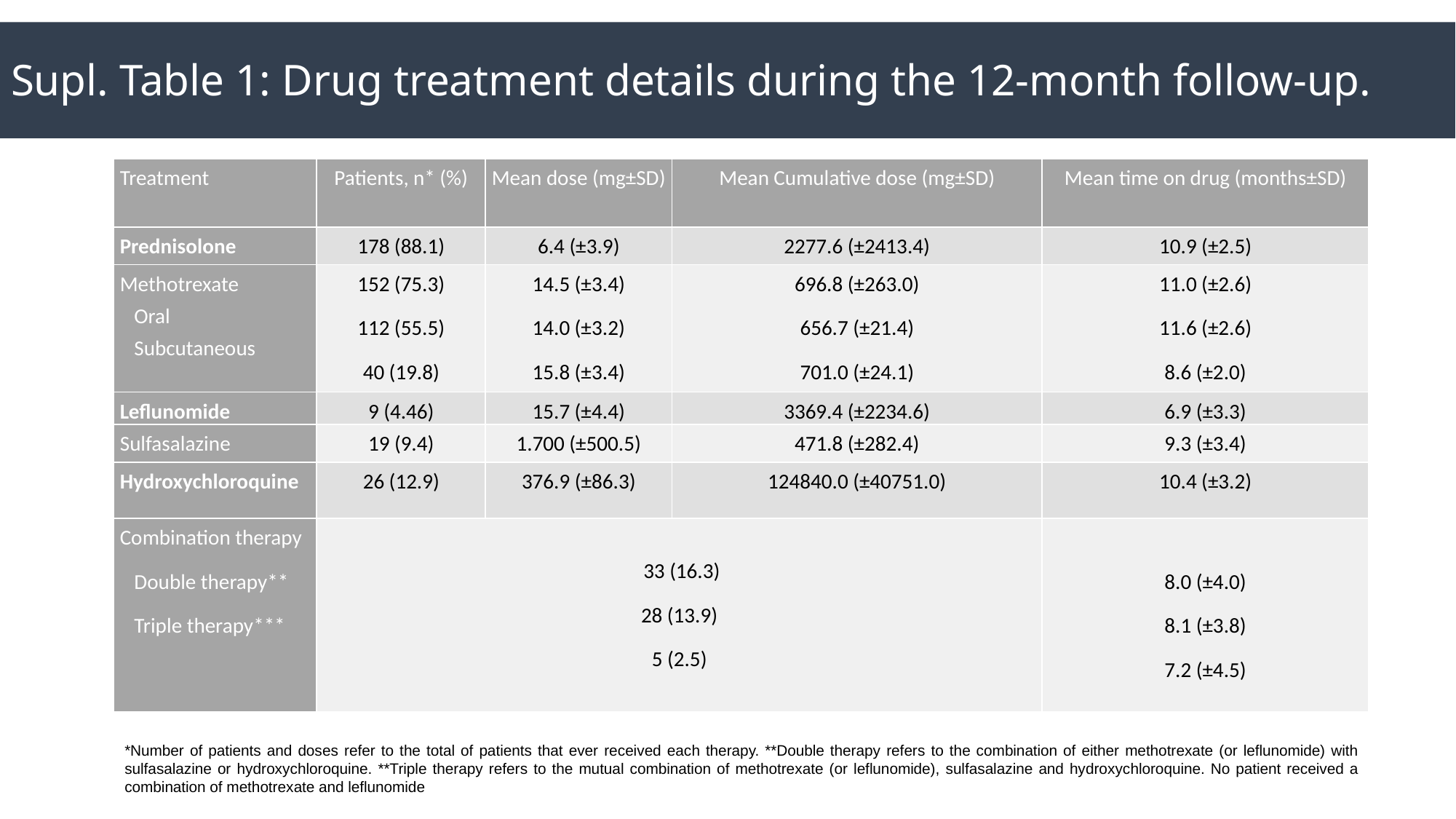

Supl. Table 1: Drug treatment details during the 12-month follow-up.
| Treatment | Patients, n\* (%) | Mean dose (mg±SD) | Mean Cumulative dose (mg±SD) | Mean time on drug (months±SD) |
| --- | --- | --- | --- | --- |
| Prednisolone | 178 (88.1) | 6.4 (±3.9) | 2277.6 (±2413.4) | 10.9 (±2.5) |
| Methotrexate Oral Subcutaneous | 152 (75.3) 112 (55.5) 40 (19.8) | 14.5 (±3.4) 14.0 (±3.2) 15.8 (±3.4) | 696.8 (±263.0) 656.7 (±21.4) 701.0 (±24.1) | 11.0 (±2.6) 11.6 (±2.6) 8.6 (±2.0) |
| Leflunomide | 9 (4.46) | 15.7 (±4.4) | 3369.4 (±2234.6) | 6.9 (±3.3) |
| Sulfasalazine | 19 (9.4) | 1.700 (±500.5) | 471.8 (±282.4) | 9.3 (±3.4) |
| Hydroxychloroquine | 26 (12.9) | 376.9 (±86.3) | 124840.0 (±40751.0) | 10.4 (±3.2) |
| Combination therapy Double therapy\*\* Triple therapy\*\*\* | 33 (16.3) 28 (13.9) 5 (2.5) | | | 8.0 (±4.0) 8.1 (±3.8) 7.2 (±4.5) |
*Number of patients and doses refer to the total of patients that ever received each therapy. **Double therapy refers to the combination of either methotrexate (or leflunomide) with sulfasalazine or hydroxychloroquine. **Triple therapy refers to the mutual combination of methotrexate (or leflunomide), sulfasalazine and hydroxychloroquine. No patient received a combination of methotrexate and leflunomide

## Slide 9
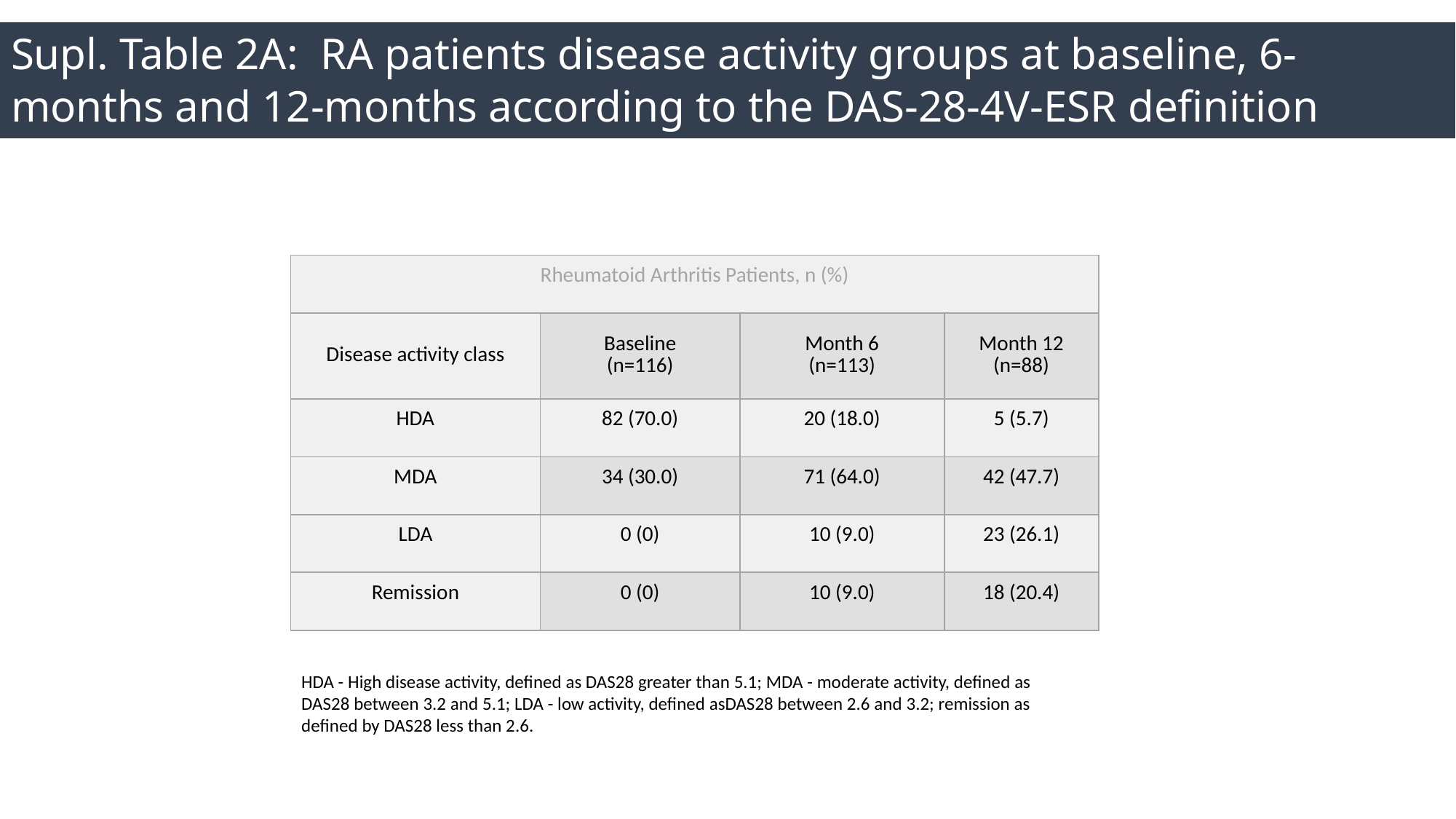

Supl. Table 2A: RA patients disease activity groups at baseline, 6-months and 12-months according to the DAS-28-4V-ESR definition
| Rheumatoid Arthritis Patients, n (%) | | | |
| --- | --- | --- | --- |
| Disease activity class | Baseline (n=116) | Month 6 (n=113) | Month 12 (n=88) |
| HDA | 82 (70.0) | 20 (18.0) | 5 (5.7) |
| MDA | 34 (30.0) | 71 (64.0) | 42 (47.7) |
| LDA | 0 (0) | 10 (9.0) | 23 (26.1) |
| Remission | 0 (0) | 10 (9.0) | 18 (20.4) |
HDA - High disease activity, defined as DAS28 greater than 5.1; MDA - moderate activity, defined as DAS28 between 3.2 and 5.1; LDA - low activity, defined asDAS28 between 2.6 and 3.2; remission as defined by DAS28 less than 2.6.

## Slide 10
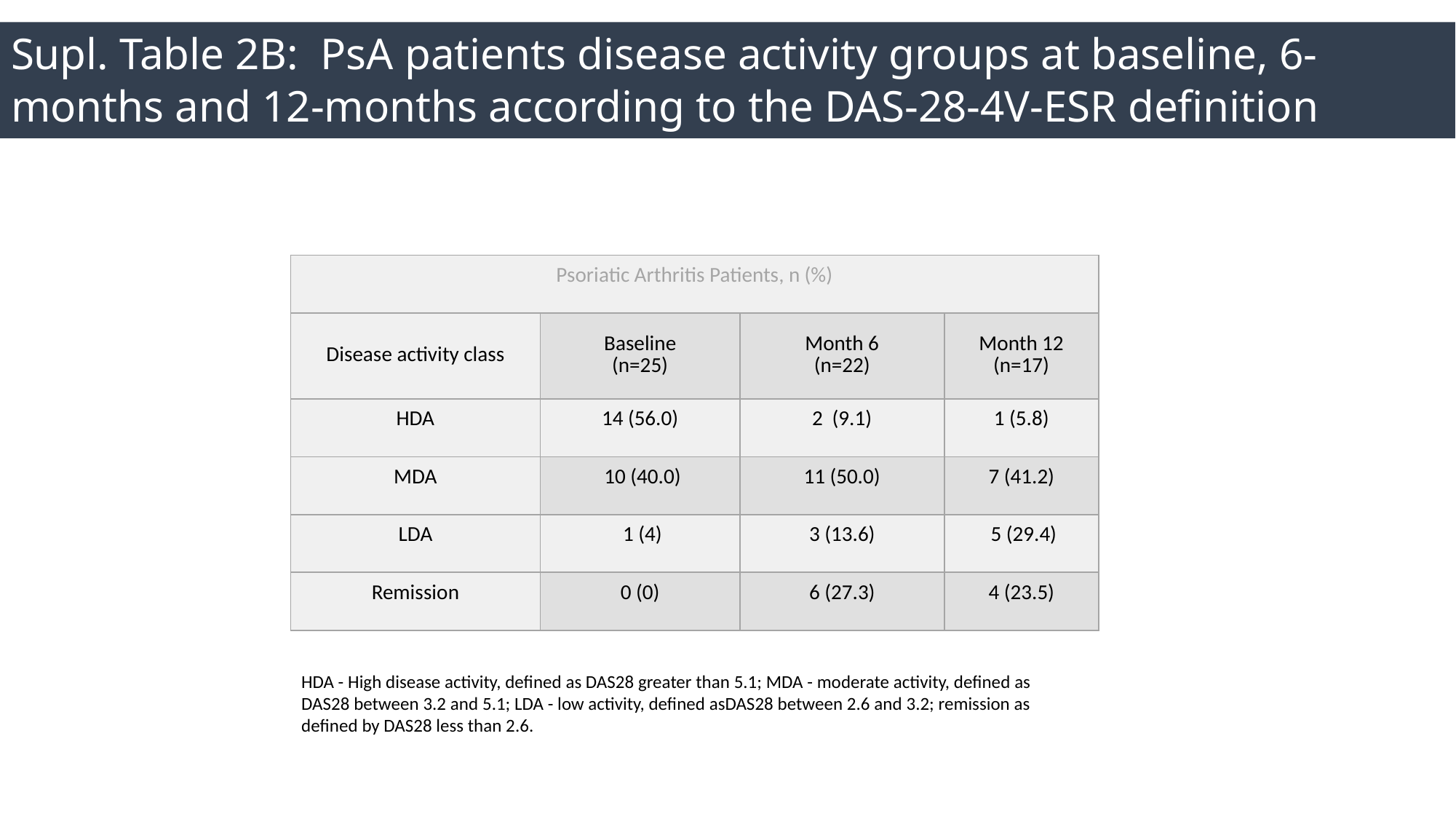

Supl. Table 2B: PsA patients disease activity groups at baseline, 6-months and 12-months according to the DAS-28-4V-ESR definition
| Psoriatic Arthritis Patients, n (%) | | | |
| --- | --- | --- | --- |
| Disease activity class | Baseline (n=25) | Month 6 (n=22) | Month 12 (n=17) |
| HDA | 14 (56.0) | 2 (9.1) | 1 (5.8) |
| MDA | 10 (40.0) | 11 (50.0) | 7 (41.2) |
| LDA | 1 (4) | 3 (13.6) | 5 (29.4) |
| Remission | 0 (0) | 6 (27.3) | 4 (23.5) |
HDA - High disease activity, defined as DAS28 greater than 5.1; MDA - moderate activity, defined as DAS28 between 3.2 and 5.1; LDA - low activity, defined asDAS28 between 2.6 and 3.2; remission as defined by DAS28 less than 2.6.

## Slide 11
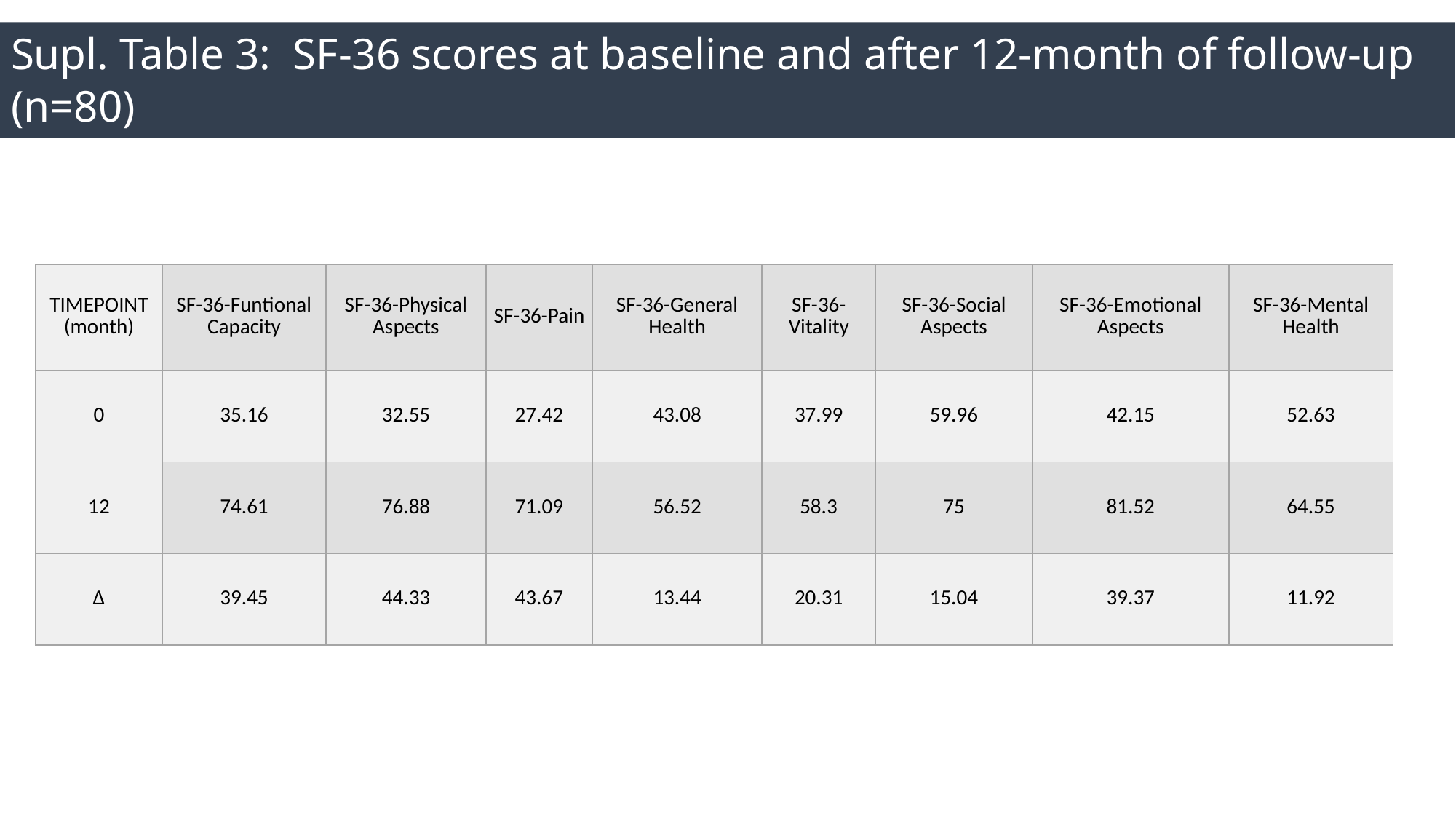

Supl. Table 3: SF-36 scores at baseline and after 12-month of follow-up (n=80)
| TIMEPOINT (month) | SF-36-Funtional Capacity | SF-36-Physical Aspects | SF-36-Pain | SF-36-General Health | SF-36-Vitality | SF-36-Social Aspects | SF-36-Emotional Aspects | SF-36-Mental Health |
| --- | --- | --- | --- | --- | --- | --- | --- | --- |
| 0 | 35.16 | 32.55 | 27.42 | 43.08 | 37.99 | 59.96 | 42.15 | 52.63 |
| 12 | 74.61 | 76.88 | 71.09 | 56.52 | 58.3 | 75 | 81.52 | 64.55 |
| Δ | 39.45 | 44.33 | 43.67 | 13.44 | 20.31 | 15.04 | 39.37 | 11.92 |

## Slide 12
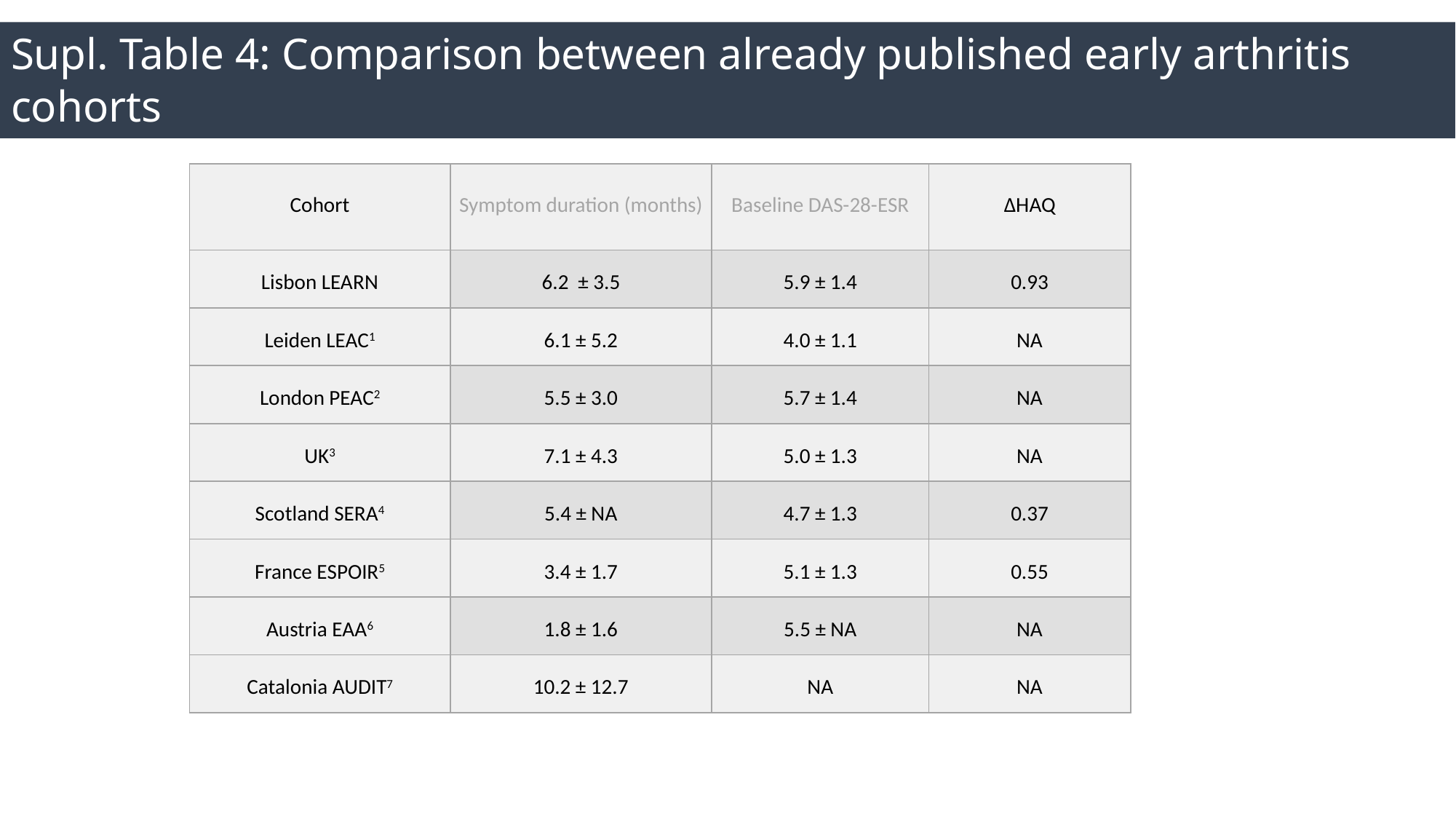

Supl. Table 4: Comparison between already published early arthritis cohorts
| Cohort | Symptom duration (months) | Baseline DAS-28-ESR | ΔHAQ |
| --- | --- | --- | --- |
| Lisbon LEARN | 6.2 ± 3.5 | 5.9 ± 1.4 | 0.93 |
| Leiden LEAC1 | 6.1 ± 5.2 | 4.0 ± 1.1 | NA |
| London PEAC2 | 5.5 ± 3.0 | 5.7 ± 1.4 | NA |
| UK3 | 7.1 ± 4.3 | 5.0 ± 1.3 | NA |
| Scotland SERA4 | 5.4 ± NA | 4.7 ± 1.3 | 0.37 |
| France ESPOIR5 | 3.4 ± 1.7 | 5.1 ± 1.3 | 0.55 |
| Austria EAA6 | 1.8 ± 1.6 | 5.5 ± NA | NA |
| Catalonia AUDIT7 | 10.2 ± 12.7 | NA | NA |

## Slide 13
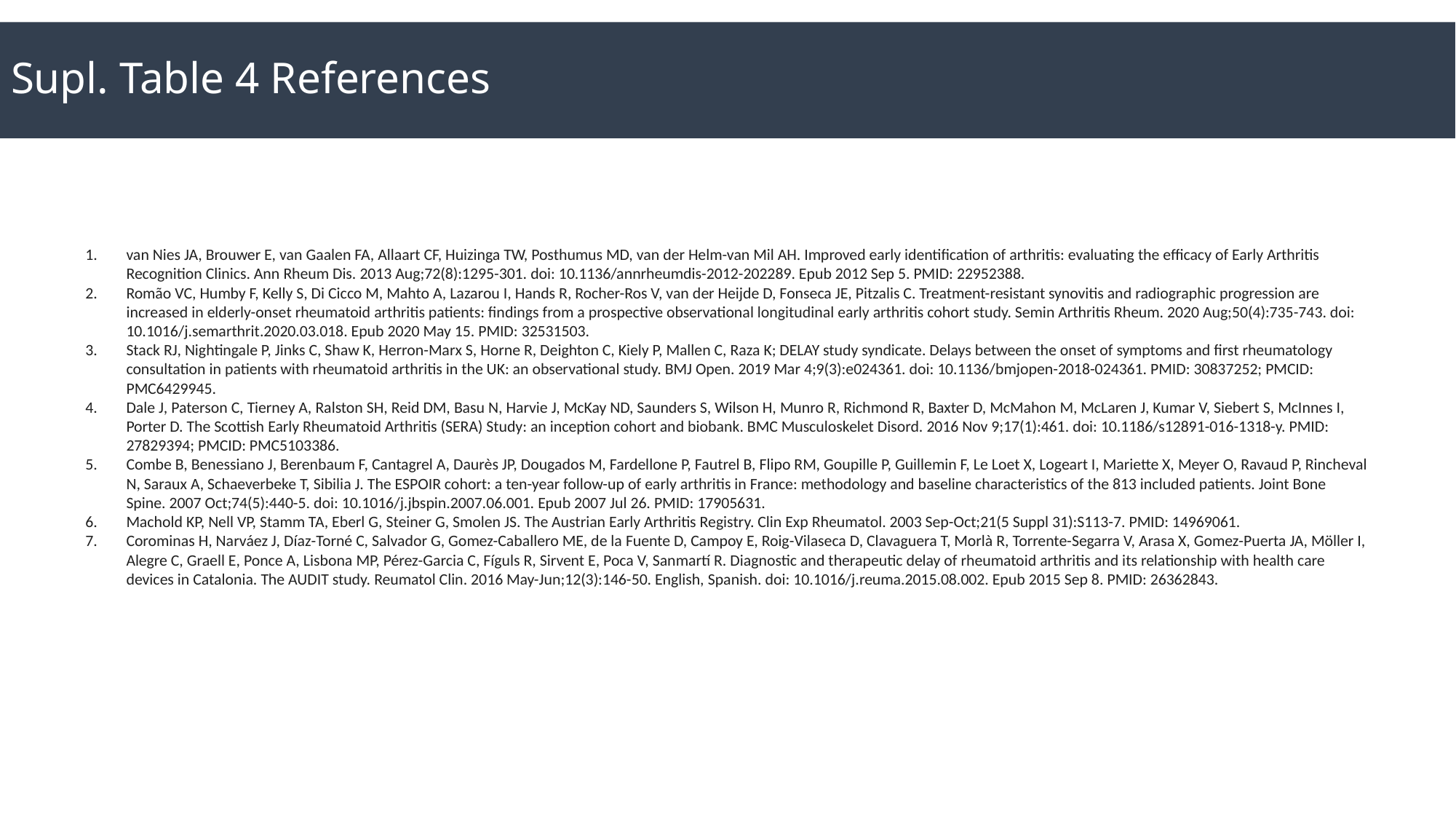

Supl. Table 4 References
van Nies JA, Brouwer E, van Gaalen FA, Allaart CF, Huizinga TW, Posthumus MD, van der Helm-van Mil AH. Improved early identification of arthritis: evaluating the efficacy of Early Arthritis Recognition Clinics. Ann Rheum Dis. 2013 Aug;72(8):1295-301. doi: 10.1136/annrheumdis-2012-202289. Epub 2012 Sep 5. PMID: 22952388.
Romão VC, Humby F, Kelly S, Di Cicco M, Mahto A, Lazarou I, Hands R, Rocher-Ros V, van der Heijde D, Fonseca JE, Pitzalis C. Treatment-resistant synovitis and radiographic progression are increased in elderly-onset rheumatoid arthritis patients: findings from a prospective observational longitudinal early arthritis cohort study. Semin Arthritis Rheum. 2020 Aug;50(4):735-743. doi: 10.1016/j.semarthrit.2020.03.018. Epub 2020 May 15. PMID: 32531503.
Stack RJ, Nightingale P, Jinks C, Shaw K, Herron-Marx S, Horne R, Deighton C, Kiely P, Mallen C, Raza K; DELAY study syndicate. Delays between the onset of symptoms and first rheumatology consultation in patients with rheumatoid arthritis in the UK: an observational study. BMJ Open. 2019 Mar 4;9(3):e024361. doi: 10.1136/bmjopen-2018-024361. PMID: 30837252; PMCID: PMC6429945.
Dale J, Paterson C, Tierney A, Ralston SH, Reid DM, Basu N, Harvie J, McKay ND, Saunders S, Wilson H, Munro R, Richmond R, Baxter D, McMahon M, McLaren J, Kumar V, Siebert S, McInnes I, Porter D. The Scottish Early Rheumatoid Arthritis (SERA) Study: an inception cohort and biobank. BMC Musculoskelet Disord. 2016 Nov 9;17(1):461. doi: 10.1186/s12891-016-1318-y. PMID: 27829394; PMCID: PMC5103386.
Combe B, Benessiano J, Berenbaum F, Cantagrel A, Daurès JP, Dougados M, Fardellone P, Fautrel B, Flipo RM, Goupille P, Guillemin F, Le Loet X, Logeart I, Mariette X, Meyer O, Ravaud P, Rincheval N, Saraux A, Schaeverbeke T, Sibilia J. The ESPOIR cohort: a ten-year follow-up of early arthritis in France: methodology and baseline characteristics of the 813 included patients. Joint Bone Spine. 2007 Oct;74(5):440-5. doi: 10.1016/j.jbspin.2007.06.001. Epub 2007 Jul 26. PMID: 17905631.
Machold KP, Nell VP, Stamm TA, Eberl G, Steiner G, Smolen JS. The Austrian Early Arthritis Registry. Clin Exp Rheumatol. 2003 Sep-Oct;21(5 Suppl 31):S113-7. PMID: 14969061.
Corominas H, Narváez J, Díaz-Torné C, Salvador G, Gomez-Caballero ME, de la Fuente D, Campoy E, Roig-Vilaseca D, Clavaguera T, Morlà R, Torrente-Segarra V, Arasa X, Gomez-Puerta JA, Möller I, Alegre C, Graell E, Ponce A, Lisbona MP, Pérez-Garcia C, Fíguls R, Sirvent E, Poca V, Sanmartí R. Diagnostic and therapeutic delay of rheumatoid arthritis and its relationship with health care devices in Catalonia. The AUDIT study. Reumatol Clin. 2016 May-Jun;12(3):146-50. English, Spanish. doi: 10.1016/j.reuma.2015.08.002. Epub 2015 Sep 8. PMID: 26362843.
